# Informatics in Undergraduate Medical Education: Analysis of Competency Frameworks and Practices Across North America

**DOI:** 10.2196/39794

**Published:** 2022-09-13

**Authors:** David Chartash, Marc Rosenman, Karen Wang, Elizabeth Chen

**Affiliations:** 1 School of Medicine University College Dublin - National University of Ireland Dublin Ireland; 2 Center for Medical Informatics Yale University School of Medicine New Haven, CT United States; 3 Ann & Robert H Lurie Children's Hospital of Chicago Chicago, IL United States; 4 Northwestern University Feinberg School of Medicine Chicago, IL United States; 5 Center for Biomedical Informatics The Warren Alpert Medical School of Brown University Providence, RI United States

**Keywords:** undergraduate medical education, medical informatics, curriculum, medical education, education, North America, framework, clinical, informatics, Canada, United States, US, teaching, management, cognitive

## Abstract

**Background:**

With the advent of competency-based medical education, as well as Canadian efforts to include clinical informatics within undergraduate medical education, competency frameworks in the United States have not emphasized the skills associated with clinical informatics pertinent to the broader practice of medicine.

**Objective:**

By examining the competency frameworks with which undergraduate medical education in clinical informatics has been developed in Canada and the United States, we hypothesized that there is a gap: the lack of a unified competency set and frame for clinical informatics education across North America.

**Methods:**

We performed directional competency mapping between Canadian and American graduate clinical informatics competencies and general graduate medical education competencies. Directional competency mapping was performed between Canadian roles and American common program requirements using keyword matching at the subcompetency and enabling competency levels. In addition, for general graduate medical education competencies, the Physician Competency Reference Set developed for the Liaison Committee on Medical Education was used as a direct means of computing the ontological overlap between competency frameworks.

**Results:**

Upon mapping Canadian roles to American competencies via both undergraduate and graduate medical education competency frameworks, the difference in focus between the 2 countries can be thematically described as a difference between the concepts of clinical and management reasoning.

**Conclusions:**

We suggest that the development or deployment of informatics competencies in undergraduate medical education should focus on 3 items: the teaching of diagnostic reasoning, such that the information tasks that comprise both clinical and management reasoning can be discussed; precision medical education, where informatics can provide for more fine-grained evaluation; and assessment methods to support traditional pedagogical efforts (both at the bedside and beyond). Assessment using cases or structured assessments (eg, Objective Structured Clinical Examinations) would help students draw parallels between clinical informatics and fundamental clinical subjects and would better emphasize the cognitive techniques taught through informatics.

## Introduction

Competency frameworks in undergraduate medical education (UME) are the key components of curricular development. Such frameworks include those developed and promulgated by internal medicine organizations and those that are used by accreditation bodies, applied in both undergraduate and graduate education. Popular competency frameworks in American UME, such as the Reporter-Interpreter-Manager-Educator framework [1], have been enhanced by commentary suggesting the addition of clinical skills relevant to the use of the electronic medical record [2]. While competency frameworks are one facet of the methods that circumscribe learning in medical education, accreditation is another facet. Accreditation serves the purposes of quality assurance and standardization. The Liaison Committee on Medical Education (LCME) standards harmonize undergraduate medical degree programs in both the United States and Canada [3]. The LCME is the primary American accreditor and a joint accreditor with the Committee on Accreditation of Medical Schools in Canada.

Standardization between the countries supports the advancement of medicine and the mobility of practice across North America. For example, as either country develops specialties, competencies can be promulgated into undergraduate and graduate medical education (GME) to best advance the general education of medical students and residents. A recent example of this phenomenon is the creation of the Canadian subspecialty of forensic psychiatry [4]. Forensic psychiatry is now a recognized specialty in both the United States and Canada, necessitating instruction alongside child and geriatric psychiatry as part of general psychiatric education in both undergraduate and GME.

In the United States, the Accreditation Council for Graduate Medical Education (ACGME) competencies for residency are a means to describe the components of the GME curriculum. These competencies span both general program requirements [5] as well as those that are specific to a particular discipline of medicine (eg, clinical informatics [6]). Similarly, supported by the Royal College of Physicians and Surgeons of Canada since 1996, Canadian medical education (both UME and GME) has developed a competency framework setting the standards for medical education and practice. The CanMEDs framework defines a set of roles for the physician, broken up into key and enabling competencies [7]. The general framework is expanded upon by the Royal College of Physicians and Surgeons of Canada or the Canadian College of Family Physicians for GME in each discipline. Furthermore, a working group from the Royal College and the Association of Faculties of Medicine of Canada has developed recommendations for nondiscipline-specific aspects of medical practice, creating sets of key and enabling competencies under the core CanMEDs roles (eg, eHealth [8]). In UME, a working group from The Association of Faculties of Medicine of Canada [9] was convened to suggest the manner in which the CanMEDs framework can be expanded to include eHealth competencies within the roles of the medical student physician. We noted that the qualifiers of “clinical,” “medical,” and “biomedical” have been applied to “informatics” as a discipline in the United States, and the broader term “eHealth” has been used in Canada. This paper selects a single term to refer to the field, *clinical informatics*, as it aligns with the formal name of the subspecialty certified by the American Board of Preventive Medicine and American Board of Pathology.

As the LCME accredits undergraduate medical programs across the North American continent, it behooves the educator to examine the Canadian approach in contrast to the American approach. Framing informatics as an additive to clinical education is contrary to Canadian efforts to integrate clinical informatics into clinical practice, be it through educational informatics (such as that developed by Ellaway et al [10,11]) or through competencies within UME. Particularly, the latter is most visible through an enabling competency within the role of *Leader* (1.4): “use health informatics to improve the quality of patient care and optimize patient safety.” If a goal of medical education is to have a unified general medical curriculum across the schools served by the LCME, it is necessary to spark a conversation about how to reconcile the Canadian role-based framework with that of the American competencies and practice. Furthermore, in reconciling the Canadian and American frameworks, we propose the beginning of an answer to the overarching question of which components of clinical informatics should be taught within UME.

## Methods

### Phase 1: Mapping of Common Program Requirements and Physician Roles

#### Overview

Mapping by keyword and content similarity was performed by human judgment of a single author (DC) and adjudicated by the remaining authors (EC, MR, and KW). Enabling competencies and key competencies were selected as the hierarchical levels at which the CanMEDs roles were to be linked to the ACGME competencies. From the 2015 CanMEDs taxonomic framework, enabling competencies are defined as the “essential components of a key competency,” while key competencies are defined as “knowledge, skills, and attitudes of a physician.” Enabling competencies are 2 hierarchical competency layers below the CanMEDs roles and link to the ACGME framework’s subcompetencies, at 1 hierarchical layer below the core competencies. The ACGME subcompetencies expand the core competencies beyond common program requirements. All maps were visualized using the *graphviz* drawing tools (using the *circo* filter; version 2.40.1; 20161225.0304).

#### Phase 1a: Mapping of Common Program Requirements and Physician Roles by Physician Competency Reference Set

Mapping was also performed using the overlap between the 2005 CanMEDs roles and the ACGME 2013 common program requirements. Instead of using keywords and content, this mapping was facilitated based on the quantization of the Physician Competency Reference Set (PCRS) [12], an ontology to which program competencies are submitted to the LCME. The PCRS is a common taxonomy of competencies used by the LCME such that multiple curricular systems across the LCME can be organized and connected through a common standard [12,13]. Individual CanMEDs enabling competencies and ACGME subcompetencies are assigned one or more PCRS concepts upon submission to the LCME, and the exemplar set provided by the LCME was used to construct the matrix of the overlap computed by the exact match of competencies mapped to each enabling competency or subcompetency.

### Phase 2: Mapping of Clinical Informatics and eHealth Competencies

Mapping was performed between the CanMEDs eHealth key competencies and the ACGME clinical informatics subcompetencies using keywords and content in a manner consistent with the human-adjudicated approach in phase 1.

### Ethics Approval

All data collected for analysis in this paper was obtained from publicly available web resources, and therefore did not necessitate review by an ethics board for any institutions affiliated by this study.

## Results

### Phase 1: Mapping of Common Program Requirements and Physician Roles

#### Overview

**Figure 1:**
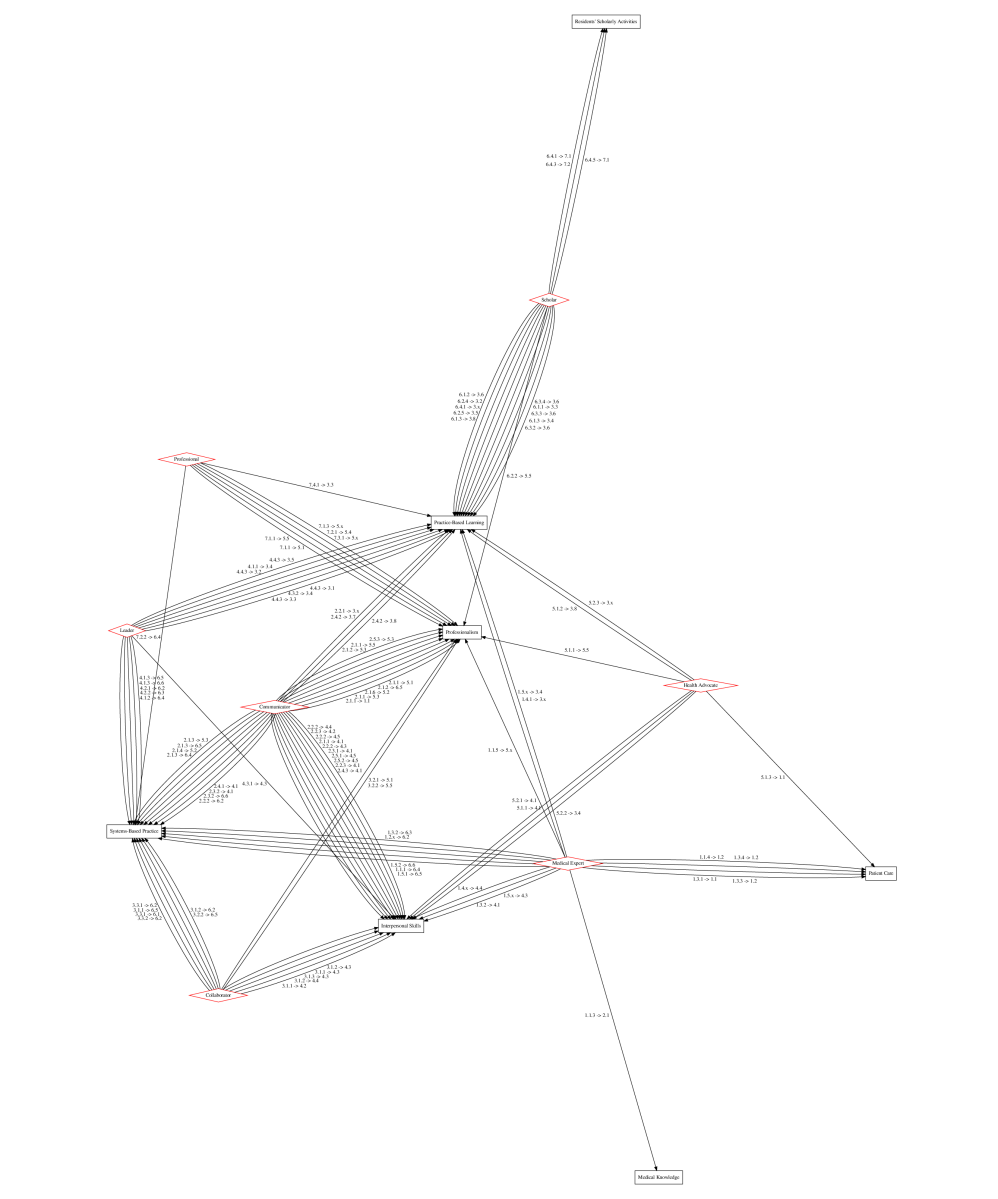
Explicit subcompetencies or enabling competencies map between CanMEDs roles through general enabling competencies [7] and Accreditation Council for Graduate Medical Education (ACGME) common program requirement core competencies [5]. CanMEDs roles are red diamonds, while ACGME competencies are in black boxes, with directional mapping labels. Higher-resolution version of this figure is available in Multimedia Appendix 1.

**Figure 2:**
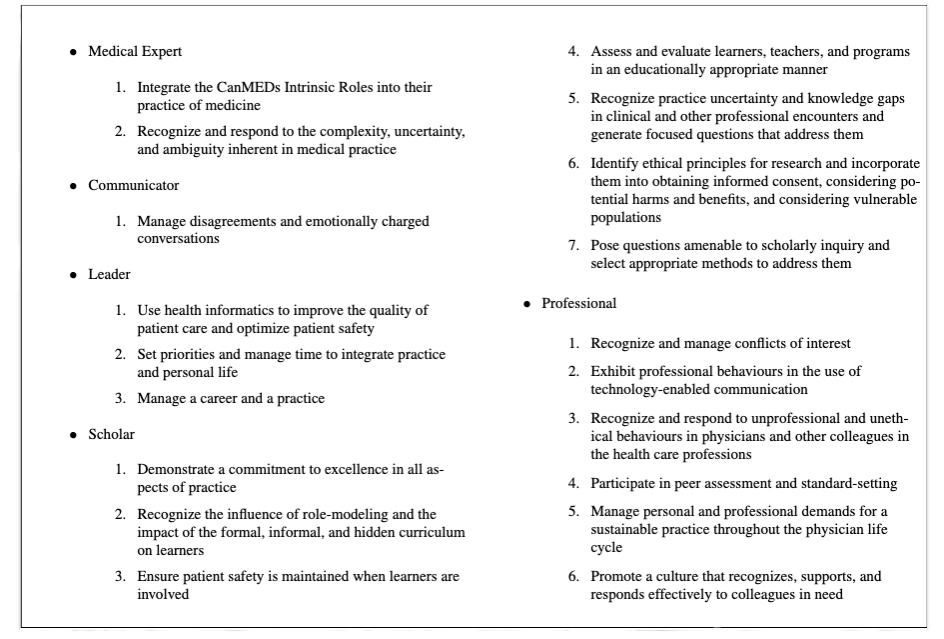
CanMEDs enabling competencies that are unmapped to Accreditation Council for Graduate Medical Education subcompetencies. Text-based version of this figure is available in Multimedia Appendix 2.

Figure 1 is a graph of the enabling competency and subcompetency map between the CanMEDs general competencies and ACGME common program requirements. Competencies are mapped by keywords or by content, with 2 examples as follows:

- Keywords: the enabling competency within the role of *health advocate* of “Incorporate disease prevention, health promotion, and health surveillance into interactions with individual patients” is mapped to a subcompetency within the competency called *patient care and procedural skills* of “Residents must be able to provide patient care that is compassionate, appropriate, and effective for the treatment of health problems and the promotion of health.” This mapping is via the keywords: “incorporate [...] health promotion” and “provide patient care that is [...] effective for [...] the promotion of health.”
- Content: the enabling competency within the role of *scholar* of “engage in collaborative learning to continuously improve personal practice and contribute to collective improvements in practice” is mapped to a subcompetency within the competency of *practice-based learning* called “participate in the education of patients, families, students, residents and other health professionals.” This mapping is based on the similarity between the concept of collaborative learning to improve personal and collective practice, and the concept of participating in the education of students, residents, and other health professionals. While collaborative learning and education are fundamentally not the same concept lexically, they are part of the tasks and practices of teaching broadly. Furthermore, while collaborative learning to collectively improve practice is centered around the individual physician in the CanMEDs role, the act of educating medical students, residents, and other health professionals consists of a similar function: improving practice by improving the care delivered by trainees.
  Figure 2 details the enabling competencies from CanMEDs roles that are unmapped to the subcompetencies of the ACGME competencies.

#### Phase 1a: Mapping of Common Program Requirements and Physician Roles by the PCRS

**Figure 3:**
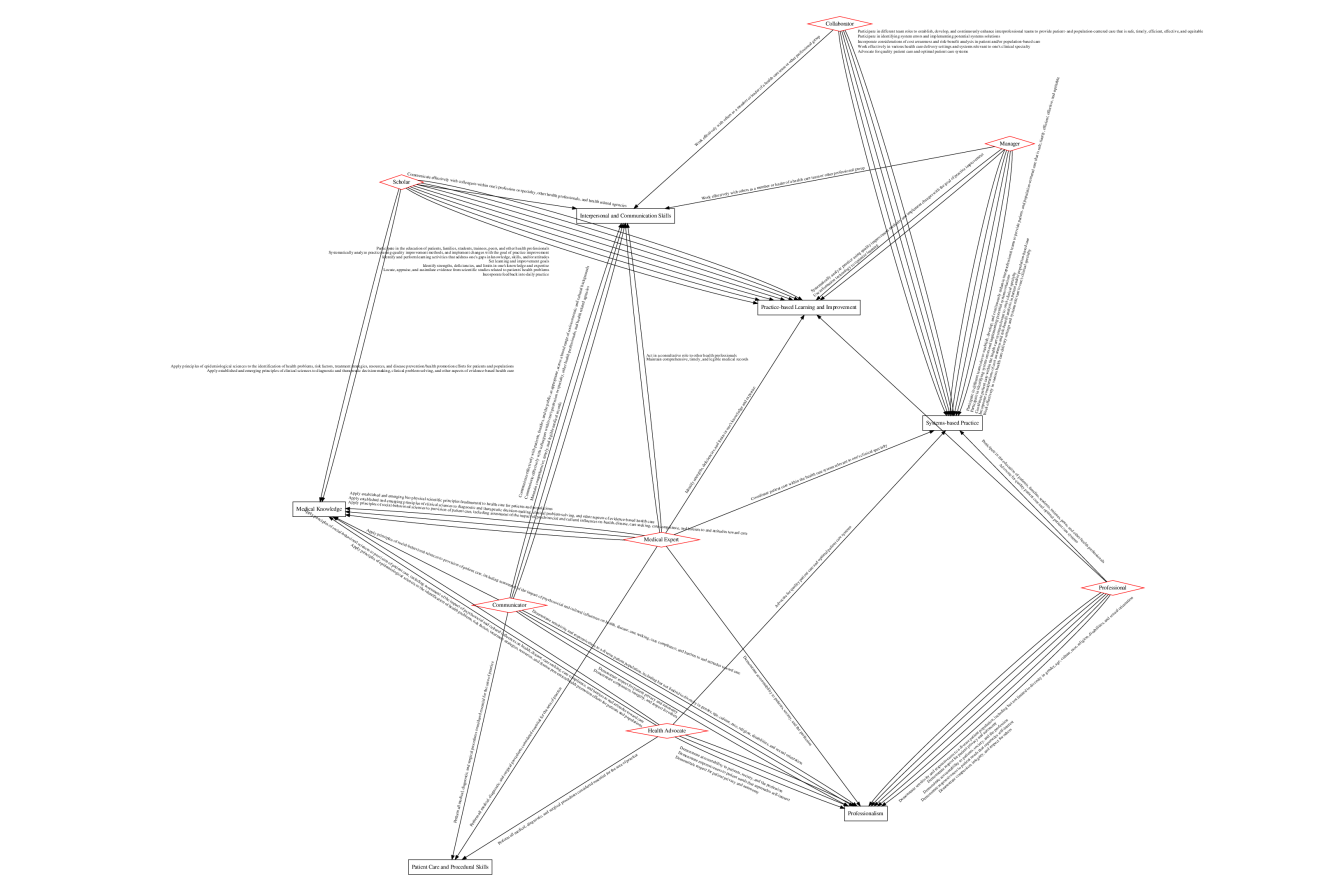
Exact graphical map between CanMEDs 2005 roles through general enabling competencies [7] and Accreditation Council for Graduate Medical Education (ACGME) 2013 common program requirement core competencies [5] detailing the Physician Competency Reference Set competencies. CanMEDs roles are red diamonds, while ACGME core competencies are in black boxes, with directional mapping labels. Higher-resolution version of this figure is available in Multimedia Appendix 3.

Figure 3 provides the direct match between the 2005 CanMEDs roles and 2013 ACGME competencies based on their mapping to the PCRS. Each edge is qualified by the PCRS competency, which is mapped to both the role and competency and is directionally assigned from the CanMEDs role to the ACGME competency. As data were available from the Association of American Medical Colleges only for the previous iterations of the CanMEDs roles and ACGME competencies, this method does not present a truly current mapping; however, it provides a method based on a standardized competency framework that we can compare with our own keywords and content approach described earlier. For example, the role of *scholar* is mapped to the competency of *practice-based learning* through the PCRS competency of “locate, appraise, and assimilate evidence from scientific studies related to patients’ health problems.” The role of *health advocates* is related to the competency of *patient care and procedural skills* through the PCRS competency of “Perform all medical, diagnostic, and surgical procedures considered essential for the area of practice.”

As a means of validation, while PCRS mapping is not equivalent to the mapping that we derived, we can examine the most and least frequent role-competency maps as a way to explore core similarities in practice between the CanMEDs and ACGME frameworks. Similar to the author-derived network in Figure 1, a comparison of the frequency of edges between CanMEDs roles and ACGME competencies in the PCRS mapping shows that the connection from *scholar* to *practice-based learning* is the most frequent. At the opposite end of the spectrum, *health advocates* and *patient care and procedural skills* were mapped only once in both graphs.

For *scholar* to *practice-based learning*, the mapping is via a subcompetency, under the competency of *practice-based learning*, called “locate, appraise, and assimilate evidence from scientific studies related to their patients’ health problems,” as well as via the CanMEDs role of *scholar* with the enabling competency of “Identify opportunities for learning and improvement by regularly reflecting on and assessing their performance using various internal and external data sources.” For *health advocate* to *patient care and procedural skills*, the mapping is via a subcompetency, under the competency of *patient care and procedural skills*, called “competently perform all medical, diagnostic, and surgical procedures considered essential for the area of practice” and via the enabling competency under the role *health advocate* called “identify the health needs of an individual patient.” *Scholar* to *practice-based learning* is a clear case of keyword mapping, *and health advocate* to *patient care and procedural skills* is more of a case of fuzzy conceptual mapping. The identification of the health needs of an individual patient is a component of performing essential procedures, such as components of the entrustable professional activities of “Form Clinical Questions and Retrieve Evidence to Advance Patient Care” and “Collaborate as a Member of an Interprofessional Team” [14]. The conceptual mapping here is deeper than the keyword overlap of “performing all essential procedures for practice” due to the circumscription of an area of practice and the determination of essential procedures (both part of the entwined acts of clinical cognition and discourse).

**Figure 4:**
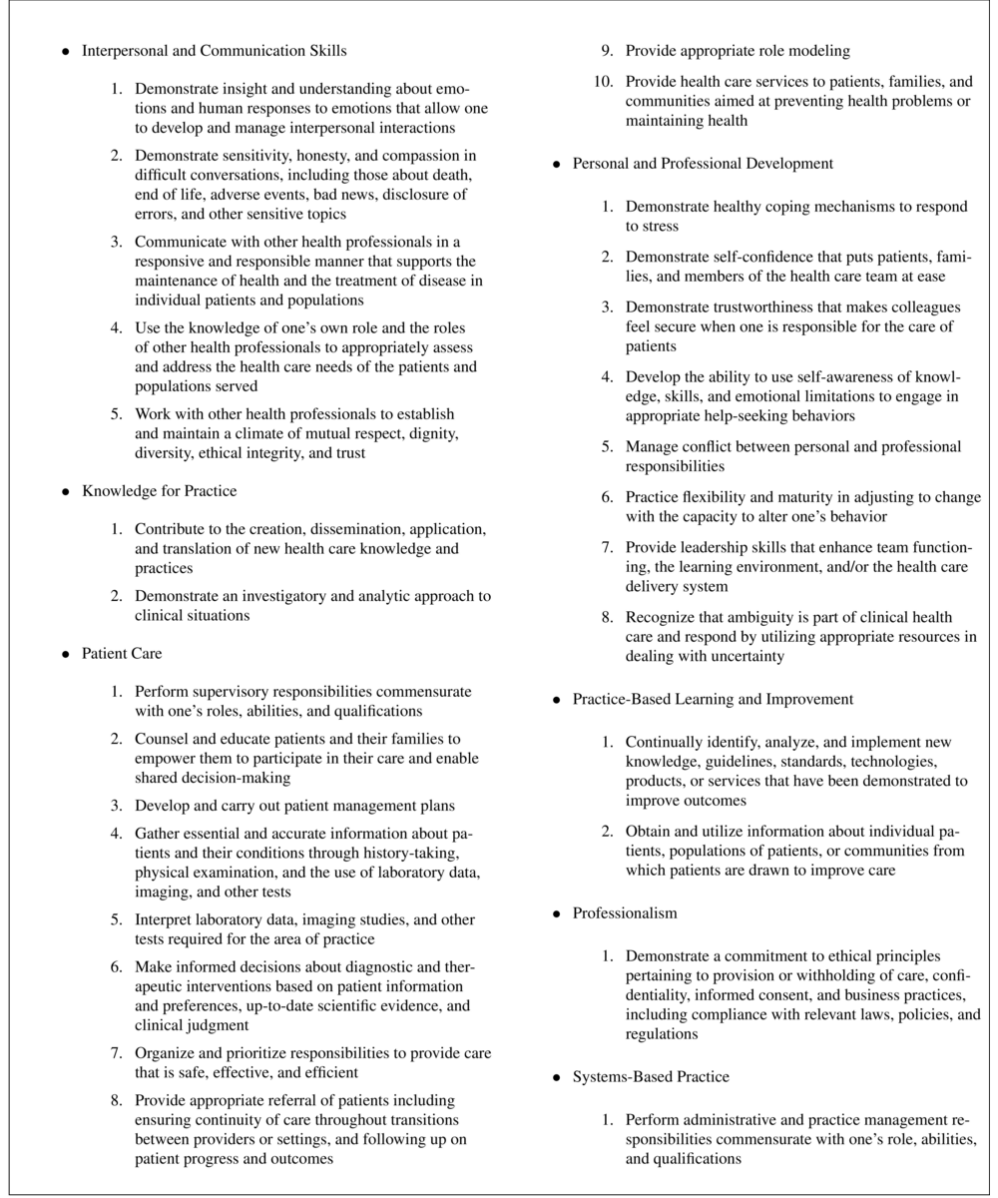
Physician Competency Reference Set (PCRS) competencies that are not mapped to either CanMEDs enabling competencies or Accreditation Council for Graduate Medical Education (ACGME) subcompetencies such that roles and competencies share a PCRS competency. Text-based version of this figure is available in Multimedia Appendix 4.

Figure 4 details the PCRS competencies that are not mapped between the CanMEDs roles and ACGME competencies. They primarily involve the comportment and ethic of the individual physician’s practice (such as “demonstrate trustworthiness that makes colleagues feel secure when one is responsible for the care of patients” or “recognize that ambiguity is part of clinical health care and respond by utilizing appropriate resources in dealing with uncertainty”). While some of these unmapped PCRS competencies are components of the aforementioned entrustable professional activities (such as trustworthiness), others are unique aspects of the CanMEDs or ACGME frameworks (such as the *medical expert* enabling competency to “recognize and respond to the complexity, uncertainty, and ambiguity inherent in medical practice”), which produce the unique cultures of medicine as practiced in Canada and the United States. However, fundamentally, these unmapped competencies reflect necessary qualities and responsibilities of the physicians.

### Phase 2: Mapping of Clinical Informatics and eHealth Competencies

**Figure 5:**
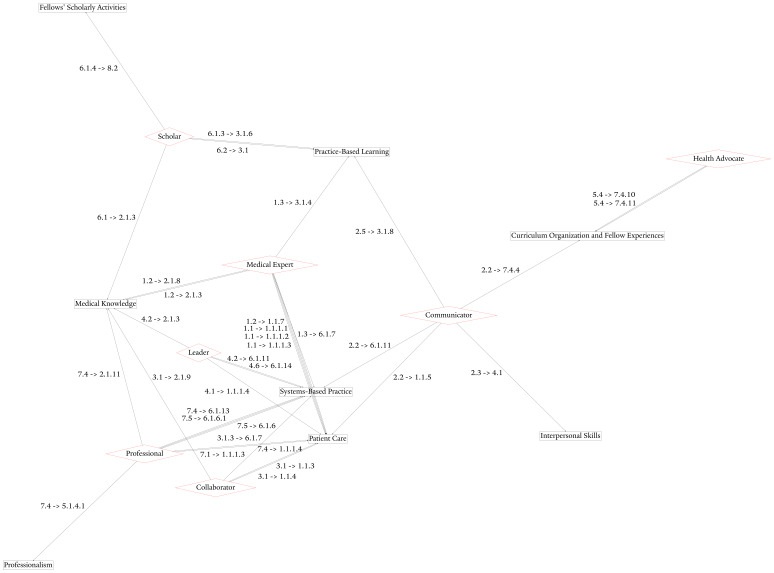
Explicit key competencies to subcompetencies map between CanMEDs eHealth Roles [8] and Accreditation Council for Graduate Medical Education (ACGME) clinical informatics core competencies [6]. CanMEDs roles are red diamonds, while ACGME core competencies are in black boxes, with directional mapping labels. Higher-resolution version of this figure is available in Multimedia Appendix 5.

Figure 5 shows a graphical representation of the specific key competencies and subcompetencies mapped between the CanMEDs roles and ACGME competencies. Competencies are mapped by keywords or content, with the following 2 examples:

- Keywords: the CanMEDs key competency for the role of *medical expert*, “employ clinical decision support tools as an adjunct to clinical judgment in providing timely, evidence-based, safe interventions,” maps based on keywords (clinical decision support and interventions or implementation) to the ACGME subcompetency of *medical knowledge* called “must demonstrate knowledge of clinical decision design, support, use, and implementation.”
- Content: the CanMEDs key competency for the role of *health advocate*, “describe how health and population information can be used for disease surveillance, adverse event tracking, population health monitoring, and risk management,” has a link to the ACGME subcompetency within the *curriculum organization and fellow experiences* core competency called “educational assignments should have a particular focus (or foci), such as: public health informatics.” Conceptually, there is a link through the domain of public health informatics and its operationalization of disease surveillance, adverse event tracking, and population health as a matter of praxis.

**Figure 6:**
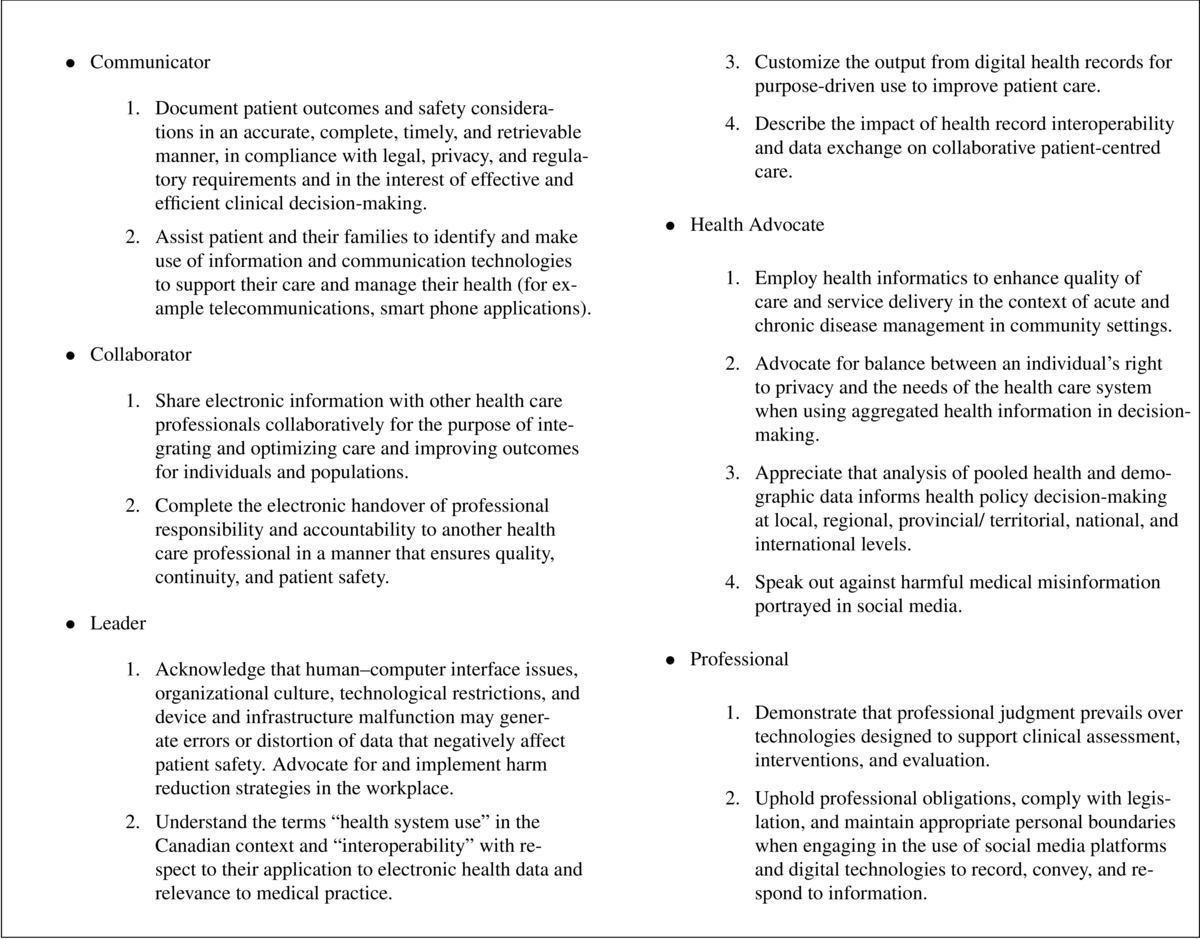
CanMEDs eHealth enabling competencies that are unmapped to Accreditation Council for Graduate Medical Education clinical informatics subcompetencies. Text-based version of this figure is available in Multimedia Appendix 6.

Figure 6 details the enabling competencies from CanMEDs eHealth roles that are unmapped to the subcompetencies of the ACGME clinical informatics competencies.

## Discussion

### History of UME in Clinical Informatics

Early efforts to integrate clinical informatics into UME hit a tipping point in 1998 with the Medical School Objectives Project (MSOP) [15]. The MSOP suggested the integration of technical skills related to the storage, retrieval, and management of information for medical problem-solving and decision-making. More recently, efforts to integrate clinical informatics into UME have used the ACGME clinical informatics competencies as a means to articulate how informatics can help prepare medical students for residency and beyond. Hersh et al [16] note that the competencies required for clinical informatics go beyond the simple information retrieval tasks described in the MSOP and that the definition of clinical informatics has evolved beyond solely the “how, what, when or why of information use” for problem-solving and clinical decision-making. Clinical informatics is not alone as a subspecialty whose content is a useful addition to UME. Other facets of the MSOP, such as public health, epidemiology, and medical ethics, have been suggested as useful additions to UME, most recently under the umbrella of health systems science [17-20]. However, it is important that when adding to the UME curriculum, medical schools attend to the need to integrate additive content into that of clinical medicine in a manner that supports the development of skills central to medicine: diagnosis and medical decision-making [21].

The use of the ACGME competencies to inform curricular elements was demonstrated by Silverman et al [22] and Hersh et al [23]. These and other previous efforts to integrate clinical informatics into UME curricula at a school level have resulted in a curriculum patterned after 2 sources: the graduate competencies for clinical informatics developed for the ACGME [6] and the core content for clinical informatics developed by the American Medical Informatics Association [24]. In these contexts, informatics serves as an integrating component at the nexus of the domains of clinical care, the health system, and information and communications technology. The resulting categories of clinical informatics content derived from the ACGME competencies were as follows: (1) fundamentals (basic knowledge and common vocabulary for the discipline), (2) clinical decision-making and care process improvement (for the implementation of systems and development of processes supporting clinical care), (3) health information systems (for the development or selection of information systems), and (4) leadership and management of change (in the implementation of clinical information systems). In an effort to refresh the core content of informatics to meet the current generation of technology, and to work toward rewriting the ACGME competencies and core content, Silverman et al [25] performed a practice analysis and developed a new set of categories (domains): (1) fundamental knowledge and skills, (2) improving care delivery and outcomes, (3) enterprise information systems, (4) data governance and data analytics, and (5) leadership and professionalism. This refresh adds a category (data governance and analytics), as well as modifies the prior focus on clinical decision-making and care process improvement. Furthermore, the shift from decision support as a core category in clinical informatics to a more generalizable focus on systems and processes is a crucial inflection point for the emergence of the discipline of clinical informatics. This shift in clinical informatics broadens its emphasis to include methods or applications work rather than focus on the fundamental clinical problems of medical decision-making. Clinical informatics is now broader than medicine and encompasses the formalized subdisciplines of nursing informatics, public health informatics, and health informatics, and it loses the focus on clinical problem-solving with the absence of a practice focus in medicine. As is alluded to with the inclusion of clinical informatics as a facet of medical education reform within the health systems sciences, this shift further separates informatics from medicine and moves it into a curricular space alongside public health, epidemiology, and medical ethics, purely additive elective components of medical education.

### Mapping

Evaluating the mapping, including the detailed unmapped content, we observe that the Canadian competencies focus on physician responsibilities (to both self and patient) in clinical practice, while the American competencies focus on the managerial aspects of medical practice. The American clinical informatics competencies focus on the management and operation of clinical data and information, rather than on patient-facing technology and interactions. The informatics distinction that can be made would be between learning to use a clinical decision support tool rather than having knowledge of the information and data that went into the design and operation of the tool. There are 2 fundamental types of reasoning: clinical or diagnostic, and management. Clinical or diagnostic reasoning is “the integration of clinical information, medical knowledge, and contextual (situational) factors to make decisions about medical care,” whereas management reasoning is the “process of making decisions about patient management, including choices about treatment, follow-up visits, further testing, and allocation of limited resources” [26]. The decision-making emphasized in the American competencies is less about “medical care,” and more about patient management. For example, the American *patient care and procedural skills* competency is to “use informatics tools to improve assessment, interdisciplinary care planning, management, coordination, and follow-up of patients.” The Canadian *medical expert* competency is to “adopt a variety of information and communication technologies to deliver patient-centred care and provide expert consultation to diverse populations in a variety of settings.” The tasks involving management reasoning (management, care planning, coordination, and follow-up) differ somewhat from those tasks for direct patient care (consultation and patient-centered care). An integrative solution would help reconcile the differences between the American and Canadian framings of clinical informatics. Teaching both management and clinical reasoning methods would best serve the current model of reasoning in medicine (suggested by Patel and Bergl [27]), given the inherent complexity of the contextual processes girding medical art and science. The need for such reconciliation is evident in the noted difference between the countries’ clinical documentation, where the semantic value of information in the American medical record is driven by compliance and reimbursement rather than by essential clinical information [28].

In thinking toward a better solution for clinical informatics education during undergraduate medical education, it is worth mentioning that a concern raised in curricular deployment is the applicability of the content to clinical practice when it is delivered in the preclerkship curriculum [29]. Curriculum design at multiple universities has resulted in either a threaded or entangled curriculum emphasizing clinical informatics [22,23] or clerkship electives focused on clinical informatics [30]. With entangled curricula, schools typically emphasize epidemiologic or decision science principles that drive the scientific fundamentals of informatics practice, rather than an elective’s emphasis on operations and clinical decision support. In addition, when attempting to teach quality improvement (aspects of which are components of the ACGME clinical informatics competencies) during the preclerkship curriculum, informatics has focused on issues of salience to students rather than on fundamental clinical value [31].

### Next Steps

Fundamentally, integrating sequenced clinical informatics content to provide experiential engagement beyond the classroom (eg, connecting terminology and standards to other clerkship rotations) and within the academic health sciences center offers a means to ensure that informatics education is not overshadowed by clinical education [32]. We acknowledge the potential challenges of reconciling Canadian and American competencies, both at the political scale of 2 national medical education schemes as well as given the oversight of the LCME. However, as a starting point, future informatics education approaches should integrate both clinical and management reasoning and should emphasize that informatics supports the pragmatic components of clerkship education that reinforce the practice and art of doctoring.

Furthermore, within clinical informatics education, there is a lack of focus on clinical judgment and meta-cognition, both educational outcomes of clinical informatics beyond those of computer use and simple information retrieval [16]. Resolving this lack is crucial, given that in a systematic review of informatics education interventions in medical education, students have been shown to be inadequately trained on extracting, aggregating, or visualizing clinical data, leading to deficits in their practice as physicians looking to work with the electronic medical record [33]. As such, informatics training beyond that initially outlined from existing efforts such as the MSOP (ie, general computer use) is necessary. We recommend the following ways to best support future curricular development:

1. Focus clinical and informatics education on the teaching of diagnostic reasoning such that management and clinical or diagnostic reasoning can be discussed in the context of the clinical encounter and health system.
2. Develop precision medical education such that informatics-supported educational outcomes can be encouraged (and assessed) via health systems science, distributed learning, and other technology-supported pedagogical platforms [11,34,35].
3. Translate clinical and clinical informatics skills into clinical practice through an objective structured clinical examination or other structured assessments familiar to students, such that the formal assessment of informatics training can occur by a method parallel to that of clinical skills, ultimately creating cognitive and pedagogical links between the two for both graduate and postgraduate education.

In teaching reasoning, be it clinical or management-based, an understanding of how physicians shape it in practice is crucial. The curriculum should ensure that the rigor of the logical practice of diagnosis (through differential diagnosis) is enhanced in its complexity rather than via shortcuts [36]. While the oldest empirical examples of this approach demonstrated promise [37,38] and heralded current successes [39,40], the key to these successes has not been in their technological sophistication but in their ability to teach and serve the physician’s logical calculus.

This notion to augment clinical practice is behind the suggestion by Hersh et al [41] that a necessary competency to add to clinical informatics should include the use of artificial and augmented intelligence in clinical settings (as well as an understanding of the biases in algorithmic approaches). Such an inclusion of artificial intelligence in the clinical information used for diagnosis has clear links to the notions of Sir Thomas Clifford Allbutt in his seminal work titled *A System of Medicine*. Allbutt [42] suggests that information integration is the pillar of the core acts of medicine, such as diagnosis. Articulating a next step for UME to teach clinical informatics, the focus of using the computer and the electronic medical record should be to facilitate the management of uncertainty through the imprimatur of the physician’s clinical guidance. This management of uncertainty would inform medical students that information and evidence are the symbols with which the clinical encounter is interpreted by the physician. Fundamentally, the management of uncertainty strengthens the physician’s information-based resilience, fighting against the automation of care, and a technician-executor model of physicianship [43]. With appropriate informatics education, the physician encountering technology would thereby be augmented rather than supplanted. The physician’s cognition would not be replaced by “the master algorithm” [44].

Finally, to assess this augmentation, future informatics education should align with the staged assessment mechanisms of medical education rather than those of cognitive theories of learning [45], such that the clinical translatability of the knowledge gained is first and foremost in the students’ mind. Shifting to a paradigm of data-driven education that mirrors the current approach to clinical care, effective measurement and assessment [11] are crucial in determining how the (hidden) curriculum with which physicianship is borne is not rendered obsolete by digitization. Therefore, this analysis can be concluded with a final paean to the clinical informatics community in seeking to advance medical education:

Wrap your thoughts in the cloth of logic and reasoning, such that they would slip easily between the wisps of shadow that link the disciplines of medicine and complot with the complexity of that curriculum which is hidden to render the fuller modern physician.

## Appendix

Multimedia Appendix 1 Higher resolution version of Figure 1. Explicit subcompetencies or enabling competencies map between CanMEDs roles through general enabling competencies [7] and Accreditation Council for Graduate Medical Education (ACGME) common program requirement core competencies [5]. CanMEDs roles are red diamonds, while ACGME competencies are in black boxes, with directional mapping labels.

Multimedia Appendix 2 Text-based version of Figure 2. CanMEDs enabling competencies that are unmapped to Accreditation Council for Graduate Medical Education subcompetencies.

Multimedia Appendix 3 Higher-resolution version of Figure 3. Exact graphical map between CanMEDs 2005 roles through general enabling competencies [7] and Accreditation Council for Graduate Medical Education (ACGME) 2013 common program requirement core competencies [5] detailing the Physician Competency Reference Set competencies. CanMEDs roles are red diamonds, while ACGME core competencies are in black boxes, with directional mapping labels.

Multimedia Appendix 4 Text-based version of Figure 4. Physician Competency Reference Set (PCRS) competencies that are not mapped to either CanMEDs enabling competencies or Accreditation Council for Graduate Medical Education (ACGME) subcompetencies such that roles and competencies share a PCRS competency.

Multimedia Appendix 5 Higher-resolution version of Figure 5. Explicit key competencies to subcompetencies map between CanMEDs eHealth Roles [8] and Accreditation Council for Graduate Medical Education (ACGME) clinical informatics core competencies [6]. CanMEDs roles are red diamonds, while ACGME core competencies are in black boxes, with directional mapping labels.

Multimedia Appendix 6 Text-based version of Figure 6. CanMEDs eHealth enabling competencies that are unmapped to Accreditation Council for Graduate Medical Education clinical informatics subcompetencies.

## Abbreviations

ACGME: Accreditation Council for Graduate Medical Education
GME: graduate medical education
LCME: Liaison Committee on Medical Education
MSOP: Medical School Objectives Project
PCRS: Physician Competency Reference Set
UME: undergraduate medical education

## Data Availability

The data used for this study are available on request, but all the information was obtained from publicly available sources.
