# Supplementary figures and images for: Informatics in Undergraduate Medical Education: Analysis of Competency Frameworks and Practices Across North America
Source: JMIR Med Educ. 2022 Sep 13;8(3):e39794. doi: 10.2196/39794 (PMC9516378; doi:10.2196/39794)

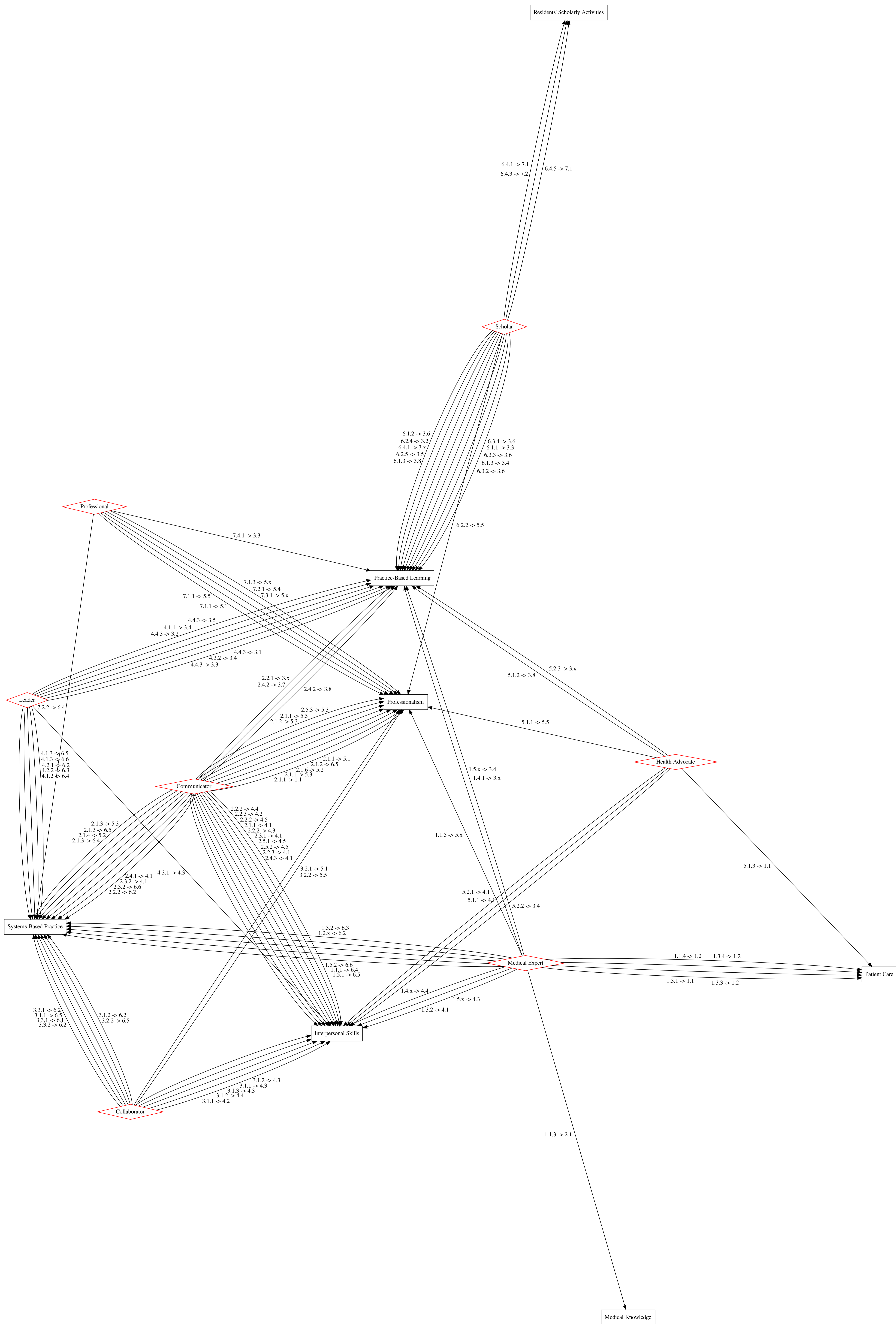

Supplement: Multimedia Appendix 1 [file mededu_v8i3e39794_app1.pdf]

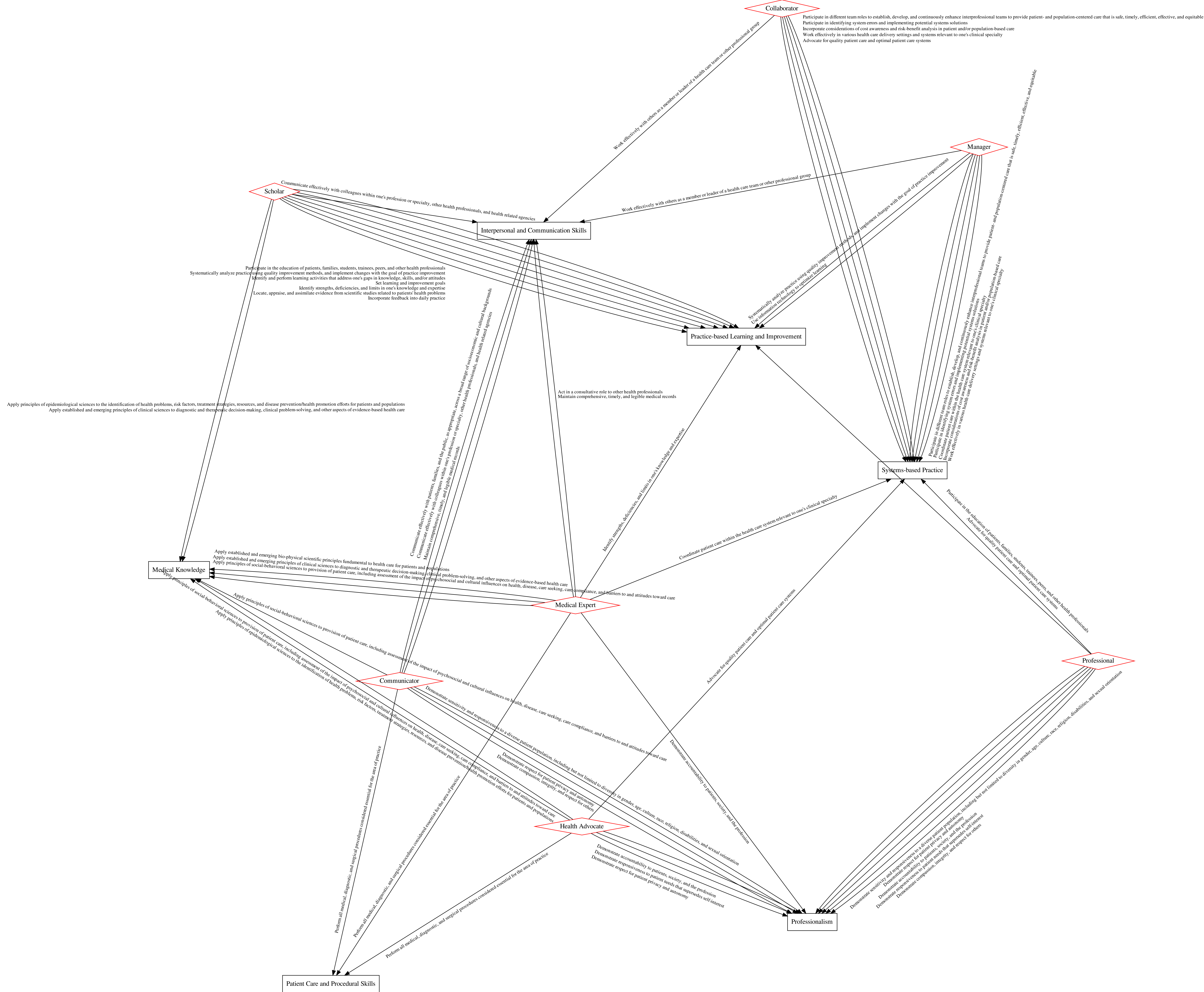

Supplement: Multimedia Appendix 3 [file mededu_v8i3e39794_app3.pdf]
