# Supplementary material for: Informatics in Undergraduate Medical Education: Analysis of Competency Frameworks and Practices Across North America
Source: JMIR Med Educ. 2022 Sep 13;8(3):e39794. doi: 10.2196/39794 (PMC9516378; doi:10.2196/39794)
Supplement: Multimedia Appendix 2 [file mededu_v8i3e39794_app2.pdf]

- Medical Expert
  1. Integrate the CanMEDs Intrinsic Roles into their practice of medicine
  2. Recognize and respond to the complexity, uncertainty, and ambiguity inherent in medical practice
- Communicator
  1. Manage disagreements and emotionally charged conversations
- Leader
  1. Use health informatics to improve the quality of patient care and optimize patient safety
  2. Set priorities and manage time to integrate practice and personal life
  3. Manage a career and a practice
- Scholar
  1. Demonstrate a commitment to excellence in all aspects of practice
  2. Recognize the influence of role-modeling and the impact of the formal, informal, and hidden curriculum on learners
  3. Ensure patient safety is maintained when learners are involved
  4. Assess and evaluate learners, teachers, and programs in an educationally appropriate manner
  5. Recognize practice uncertainty and knowledge gaps in clinical and other professional encounters and generate focused questions that address them
  6. Identify ethical principles for research and incorporate them into obtaining informed consent, considering potential harms and benefits, and considering vulnerable populations
  7. Pose questions amenable to scholarly inquiry and select appropriate methods to address them
- Professional
  1. Recognize and manage conflicts of interest
  2. Exhibit professional behaviours in the use of technology-enabled communication
  3. Recognize and respond to unprofessional and unethical behaviours in physicians and other colleagues in the health care professions
  4. Participate in peer assessment and standard-setting
  5. Manage personal and professional demands for a sustainable practice throughout the physician life cycle
  6. Promote a culture that recognizes, supports, and responds effectively to colleagues in need
