# Supplementary material for: Informatics in Undergraduate Medical Education: Analysis of Competency Frameworks and Practices Across North America
Source: JMIR Med Educ. 2022 Sep 13;8(3):e39794. doi: 10.2196/39794 (PMC9516378; doi:10.2196/39794)
Supplement: Multimedia Appendix 5 [file mededu_v8i3e39794_app5.pdf]

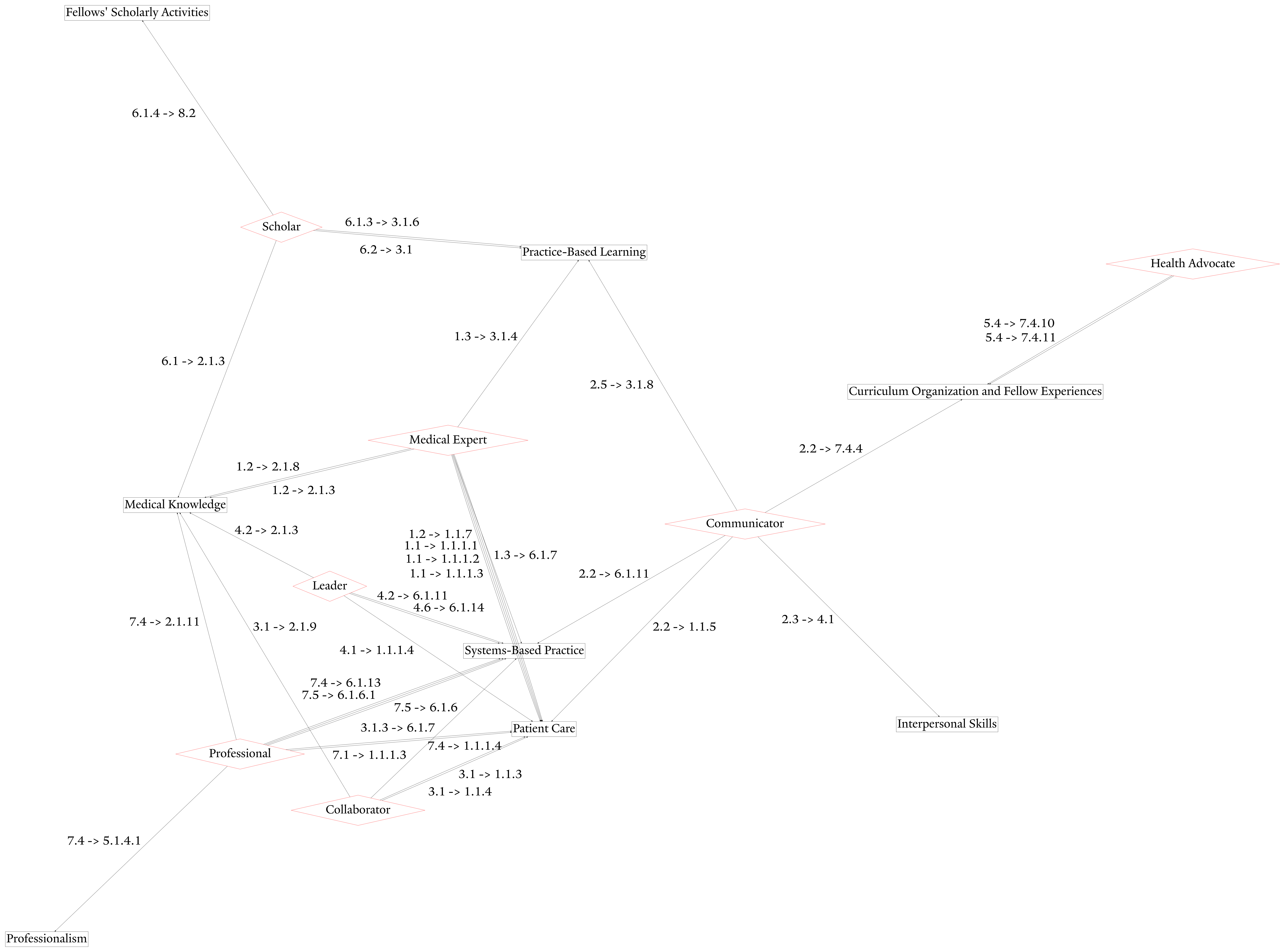
Fellows' Scholarly Activities
6.1.4 -> 8.2
Scholar
6.1.3 -> 3.1.6
6.2 -> 3.1
Practice-Based Learning
Health Advocate
5.4 -> 7.4.10
5.4 -> 7.4.11
1.3 -> 3.1.4
6.1 -> 2.1.3
2.5 -> 3.1.8
Curriculum Organization and Fellow Experiences
Medical Expert
2.2 -> 7.4.4
1.2 -> 2.1.8
1.2 -> 2.1.3
Medical Knowledge
Communicator
4.2 -> 2.1.3
1.2 -> 1.1.7
1.1 -> 1.1.1.1
1.1 -> 1.1.1.2
1.1 -> 1.1.1.3
1.3 -> 6.1.7
2.2 -> 6.1.11
Leader
4.2 -> 6.1.11
4.6 -> 6.1.14
7.4 -> 2.1.11
3.1 -> 2.1.9
2.2 -> 1.1.5
2.3 -> 4.1
4.1 -> 1.1.1.4
Systems-Based Practice
7.4 -> 6.1.13
7.5 -> 6.1.6.1
7.5 -> 6.1.6
3.1.3 -> 6.1.7
Patient Care
Interpersonal Skills
Professional
7.1 -> 1.1.1.3
7.4 -> 1.1.1.4
3.1 -> 1.1.3
3.1 -> 1.1.4
Collaborator
7.4 -> 5.1.4.1
Professionalism
