# Supplementary material for: Informatics in Undergraduate Medical Education: Analysis of Competency Frameworks and Practices Across North America
Source: JMIR Med Educ. 2022 Sep 13;8(3):e39794. doi: 10.2196/39794 (PMC9516378; doi:10.2196/39794)
Supplement: Multimedia Appendix 6 [file mededu_v8i3e39794_app6.pdf]

- Communicator

    1. Document patient outcomes and safety considerations in an accurate, complete, timely, and retrievable manner, in compliance with legal, privacy, and regulatory requirements and in the interest of effective and efficient clinical decision-making.
    2. Assist patient and their families to identify and make use of information and communication technologies to support their care and manage their health (for example telecommunications, smart phone applications).

- Collaborator

    1. Share electronic information with other health care professionals collaboratively for the purpose of integrating and optimizing care and improving outcomes for individuals and populations.
    2. Complete the electronic handover of professional responsibility and accountability to another health care professional in a manner that ensures quality, continuity, and patient safety.

- Leader

    1. Acknowledge that human–computer interface issues, organizational culture, technological restrictions, and device and infrastructure malfunction may generate errors or distortion of data that negatively affect patient safety. Advocate for and implement harm reduction strategies in the workplace.
    2. Understand the terms "health system use" in the Canadian context and "interoperability" with respect to their application to electronic health data and relevance to medical practice.
    3. Customize the output from digital health records for purpose-driven use to improve patient care.
    4. Describe the impact of health record interoperability and data exchange on collaborative patient-centred care.

- Health Advocate

    1. Employ health informatics to enhance quality of care and service delivery in the context of acute and chronic disease management in community settings.
    2. Advocate for balance between an individual's right to privacy and the needs of the health care system when using aggregated health information in decision-making.
    3. Appreciate that analysis of pooled health and demographic data informs health policy decision-making at local, regional, provincial/ territorial, national, and international levels.
    4. Speak out against harmful medical misinformation portrayed in social media.

- Professional

    1. Demonstrate that professional judgment prevails over technologies designed to support clinical assessment, interventions, and evaluation.
    2. Uphold professional obligations, comply with legislation, and maintain appropriate personal boundaries when engaging in the use of social media platforms and digital technologies to record, convey, and respond to information.
