# Supplementary material for: Informatics in Undergraduate Medical Education: Analysis of Competency Frameworks and Practices Across North America
Source: JMIR Med Educ. 2022 Sep 13;8(3):e39794. doi: 10.2196/39794 (PMC9516378; doi:10.2196/39794)
Supplement: Multimedia Appendix 4 [file mededu_v8i3e39794_app4.pdf]

- Interpersonal and Communication Skills

    1. Demonstrate insight and understanding about emotions and human responses to emotions that allow one to develop and manage interpersonal interactions
    2. Demonstrate sensitivity, honesty, and compassion in difficult conversations, including those about death, end of life, adverse events, bad news, disclosure of errors, and other sensitive topics
    3. Communicate with other health professionals in a responsive and responsible manner that supports the maintenance of health and the treatment of disease in individual patients and populations
    4. Use the knowledge of one's own role and the roles of other health professionals to appropriately assess and address the health care needs of the patients and populations served
    5. Work with other health professionals to establish and maintain a climate of mutual respect, dignity, diversity, ethical integrity, and trust

- Knowledge for Practice

    1. Contribute to the creation, dissemination, application, and translation of new health care knowledge and practices
    2. Demonstrate an investigatory and analytic approach to clinical situations

- Patient Care

    1. Perform supervisory responsibilities commensurate with one's roles, abilities, and qualifications
    2. Counsel and educate patients and their families to empower them to participate in their care and enable shared decision-making
    3. Develop and carry out patient management plans
    4. Gather essential and accurate information about patients and their conditions through history-taking, physical examination, and the use of laboratory data, imaging, and other tests
    5. Interpret laboratory data, imaging studies, and other tests required for the area of practice
    6. Make informed decisions about diagnostic and therapeutic interventions based on patient information and preferences, up-to-date scientific evidence, and clinical judgment
    7. Organize and prioritize responsibilities to provide care that is safe, effective, and efficient
    8. Provide appropriate referral of patients including ensuring continuity of care throughout transitions between providers or settings, and following up on patient progress and outcomes
    9. Provide appropriate role modeling
    10. Provide health care services to patients, families, and communities aimed at preventing health problems or maintaining health

- Personal and Professional Development

    1. Demonstrate healthy coping mechanisms to respond to stress
    2. Demonstrate self-confidence that puts patients, families, and members of the health care team at ease
    3. Demonstrate trustworthiness that makes colleagues feel secure when one is responsible for the care of patients
    4. Develop the ability to use self-awareness of knowledge, skills, and emotional limitations to engage in appropriate help-seeking behaviors
    5. Manage conflict between personal and professional responsibilities
    6. Practice flexibility and maturity in adjusting to change with the capacity to alter one's behavior
    7. Provide leadership skills that enhance team functioning, the learning environment, and/or the health care delivery system
    8. Recognize that ambiguity is part of clinical health care and respond by utilizing appropriate resources in dealing with uncertainty

- Practice-Based Learning and Improvement

    1. Continually identify, analyze, and implement new knowledge, guidelines, standards, technologies, products, or services that have been demonstrated to improve outcomes
    2. Obtain and utilize information about individual patients, populations of patients, or communities from which patients are drawn to improve care

- Professionalism

    1. Demonstrate a commitment to ethical principles pertaining to provision or withholding of care, confidentiality, informed consent, and business practices, including compliance with relevant laws, policies, and regulations

- Systems-Based Practice

    1. Perform administrative and practice management responsibilities commensurate with one's role, abilities, and qualifications
